# Mechanically Strong Polyurethane Composites Reinforced with Montmorillonite-Modified Sage Filler (*Salvia officinalis* L.)

**DOI:** 10.3390/ijms22073744

**Published:** 2021-04-03

**Authors:** Sylwia Członka, Agnė Kairytė, Karolina Miedzińska, Anna Strąkowska, Agnieszka Adamus-Włodarczyk

**Affiliations:** 1 Faculty of Chemistry, Institute of Polymer & Dye Technology, Lodz University of Technology, 90-924 Lodz, Poland; karolina.miedzinska@dokt.p.lodz.pl (K.M.); anna.strakowska@p.lodz.pl (A.S.); 2 Laboratory of Thermal Insulating Materials and Acoustics, Faculty of Civil Engineering, Institute of Building Materials, Vilnius Gediminas Technical University, Linkmenu St. 28, LT-08217 Vilnius, Lithuania; agne.kairyte@vilniustech.lt; 3 Faculty of Chemistry, Institute of Applied Radiation Chemistry, Lodz University of Technology, 93-590 Lodz, Poland; agnieszka.adamus@p.lodz.pl

**Keywords:** polyurethanes, composites, mechanical strength, thermal conductivity, flame-retardancy

## Abstract

Rigid polyurethane (PUR) foams reinforced with 1, 2, and 5 wt.% of salvia filler (SO filler) and montmorillonite-modified salvia filler (MMT-modified SO filler) were produced in the following study. The impact of 1, 2, and 5 wt.% of SO filler and MMT-modified SO filler on the morphological, chemical, and mechanical properties of PUR composites were examined. In both cases, the addition of 1 and 2 wt.% of SO fillers resulted in the synthesis of PUR composites with improved physicomechanical properties, while the addition of 5 wt.% of SO fillers resulted in the formation of PUR composites with a less uniform structure and, therefore, some deterioration in their physicomechanical performances. Moreover, the results showed that the modification of SO filler with MMT improved the interphase compatibility between filler surface and PUR matrix. Therefore, such reinforced PUR composites were characterized by a well-developed closed-cell structure and improved mechanical, thermal, and flame-retardant performances. For example, when compared with reference foam, the addition of 2 wt.% of MMT-modified SO filler resulted in the formation of PUR composites with greater mechanical properties (compressive strength, flexural strength) and improved dynamic-mechanical properties (storage modulus). The PUR composites were characterized by better thermal stability as well as improved flame retardancy—e.g., decreased peak rate of heat release (pHRR), reduced total smoke release (TSR), and increased limiting oxygen index (LOI).

## 1. Introduction

Currently, scientists around the world are focusing on the use of renewable resources in the production of polymeric materials, in line with the requirements of sustainable development [1,2,3]. New methods and the use of natural raw materials are designed to reduce the consumption of non-renewable resources and obtain new biomaterials [4,5,6,7].

The polyurethane (PUR) material was first obtained in 1849 by Wurtz. Later, in 1937, Otto Bayer found that this interesting polymer could be obtained as a reaction product of a diisocyanate and a polyol [8]. Nowadays, polyurethanes are a dynamically developing group of complex polymers. They are obtained by the reaction of compounds containing at least two active hydrogen atoms (most often polyols) with isocyanates in the presence of catalysts, surfactants, blowing agents, and other additives [9]. This is the reaction of forming a urethane bond, which is the backbone of the resulting polymer [10]. In addition to urethane linkages, depending on the components used in the synthesis, polyurethanes can contain aliphatic or aromatic hydrocarbons, amides, esters, biuret, or isocyanurate groups. Due to the wide variety of substrates and additives used during synthesis, it is possible to control the physical, mechanical and chemical properties of the finished materials [10]. Given the wide range of raw materials used, polyurethanes are applied in various forms, e.g., foams (rigid or flexible), coatings, adhesives, sealants, films, fibers, and many more [11]. This allows them to be used in a wide range of applications in many industries including construction, automotive, packaging, electronics, or furniture [12].

The growing environmental requirements and the principles of sustainable development influenced the search for new natural modifiers used in the production of polymer materials [13]. Raw materials from trees and plants play a special role as bio-fillers due to their availability and low cost of obtaining. Moreover, the interest in using natural additives was also caused by the increasing costs of storage and utilization of this type of waste. It turned out that the use of natural waste could therefore be a good solution for waste management [14]. In recent years, there was a growing interest in the application of natural additives in the form of bio-polyol and bio-fillers, especially cellulosic and lignocellulosic raw materials such as nutshells, fruit seeds, coconut fibers, extracts, and others [6,12,15,16,17,18,19,20,21].

The filler considered in this article is the extract by Salvia officinalis (commonly known as sage), without excluding the potential use of other perennial species of the genus Salvia, very similar to S. officinalis such as Salvia fruticosa subsp. thomasii [22]. Sage is an aromatic plant with a large scale of bioactive compounds, which is widely used in traditional medicine [23,24]. It is estimated that there are around 900 species of sage, some of which are used in medicine and cosmetics. Several species of this plant exhibit interesting properties, including antibacterial or antioxidant [25]. Most of the studies reported in the literature focused on the biological (i.e., hepatoprotective and nephroprotective) effects of sage and its oils [26,27]. In this article, the sage extract will be modified with montmorillonite and used as a bio-filler in the preparation of rigid polyurethane foams.

The main group of polyurethane materials, which dominates the market with over 65% of the world’s production of polyurethanes, are foams [8,28]. Moreover, it is estimated that in recent years, the global demand for polyurethane foams amounted to approximately 5% of the global consumption of plastics [29]. These are materials characterized by a porous structure, high strength, and low apparent density. Their individual properties can be modified by the appropriate selection of components used during synthesis and their mutual relations [10,30]. Depending on the material parameters, porous PUR materials can be divided into two main groups: flexible polyurethane foams (FPUFs) and rigid polyurethane foams (RPUFs). The most significant functional features of foams are the low thermal conductivity and dimensional stability, which are determined by the cellular structure. The content of the open cells in the structure increases heat transfer. Therefore, foams with a higher content of closed cells show lower values of the thermal conductivity coefficient—that is, better thermal insulation properties. Rigid polyurethane foams are strongly cross-linked materials characterized mainly by closed cell structures. As for the application, PUR foams are generally used as low-cost materials mainly in construction, as thermal insulation materials, electronics, furniture, packaging, and others [30,31].

The significant disadvantages of PUR foams are their high flammability and easy ignition. For a long time, these factors did limit their use in many engineering applications [32]. It is well known that the degradation of the urethane bonds of rigid polyurethane foams begins at 200 °C [33]. There are a few ways for improving the fire retardancy of PUR foams:

- The incorporation of flame-retardant additives, also known as anti-pyrenes [34], into the reaction mixture at the component mixing stage,
- the application of compounds that contain flame-retardant functional groups (especially hydroxyl),
- the formation of a fire-retardant coating on the surface of the flammable foam.

Various types of flame retardants are known, including: phosphorous compounds [35], bromine compounds [36], melamine compounds [37], expandable graphite [36,38], inorganic salts [39]. Moreover, inorganic metal oxides and hydroxides, including compounds of magnesium, silicon, and aluminum, also play an important role in reducing combustibility [40,41,42,43]. Halogen compounds have also been used previously as flame retardants. However, due to the increasing environmental requirements in recent times, their application is limited because of the release of toxic and harmful gases from their combustion [30,44]. That is why, in recent years, the focus was on the use of halogen-free flame retardants that do not harm the environment.

One of the commonly used flame retardants is montmorillonite (MMT), described with the chemical composition of Al_2_O_3_·4SiO_2_·3H_2_O, where the octahedron Al-O layer is between two tetrahedron Si-O layers [45]. Montmorillonite is a multi-layered hydrophilic nanofiller with a high-aspect-ratio and a large specific surface [46]. Due to its ability to create a thermal barrier and delay thermal degradation, it is widely used to increase the thermal stability and the fire resistance of polymer composites [47]. Another significant feature of montmorillonite is the ability to improve the mechanical properties of the obtained composites [48].

In the present study, the influence of used modifiers on mechanical properties and flammability of rigid polyurethane foams was assessed. Although many studies investigated the influence of various fillers on polyurethane foams, to the best of our knowledge, there was no research on PUR composites filled with modified sage filler.

## 2. Results and Discussion

### 2.1. Filler Characterization

**Figure 1:**
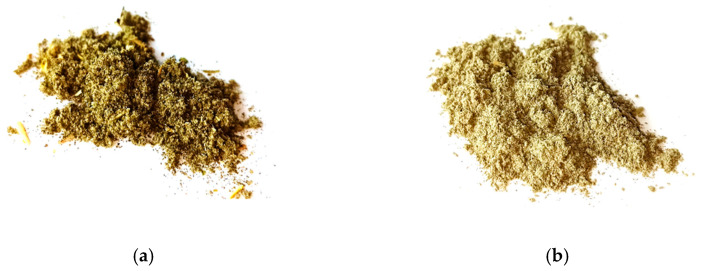
External morphology of (**a**) unmodified salvia filler (SO filler), and (**b**) montmorillonite (MMT)-modified SO filler.

**Figure 2:**
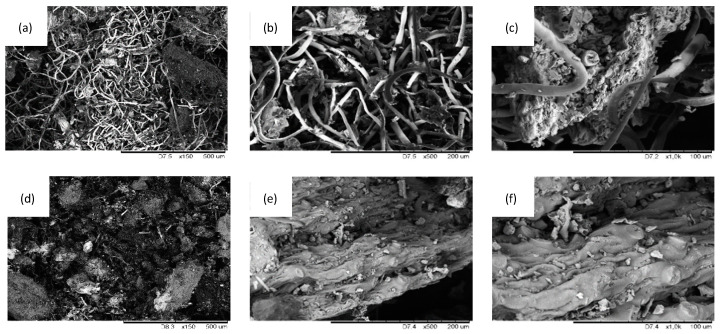
Morphological structure of (**a**–**c**) SO filler, and (**d**–**f**) MMT-modified SO filler.

The external morphology of unmodified salvia filler (SO filler) and MMT-modified SO filler is presented in Figure 1. SEM images of SO fillers are presented in Figure 2. Based on this, it may be concluded that the application of a high-energy ball milling process resulted in the formation of SO filler with smaller particles and a smoother surface. A more uniform surface of modified SO filler may be connected with the modification of SO filler with MMT, which created an external layer on the filler surface.

**Figure 3:**
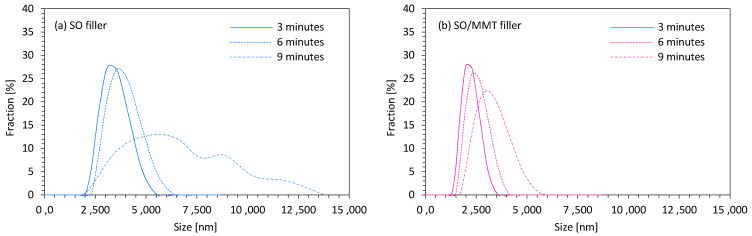
The particle size distribution of (**a**) SO filler, and (**b**) MMT-modified SO filler.

Dynamic light scattering (DLS) analysis was used to determine the size of SO filler particles. Particle size measurements were performed at 3 min intervals—after 3, 6, and 9 min from the mixing of SO fillers in the polyol system. According to the results presented in Figure 3a, the average size of unmodified SO filler was ~3.5 µm. After 9 min, the particles tended to agglomerate. The average size of filler particles significantly increased and the distribution of the particle size became less uniform. Most of the particles were in the range of 3–7 µm. Apart from this, some bigger aggregates with an average size of ~10 µm were also presented in the polyol system. According to the results presented in Figure 3b, the MMT-modified SO filler exhibited a more uniform distribution of the particles. After 3 min of filler mixing, about 30% of filler particles with an average size of ~2 µm were observed in the polyol system. After 6 min, the average size increased very slightly to ~2.5 µm. Most importantly, no significant agglomeration of filler particles was observed after 9 min. The size distribution of the particles was uniform, and most of the particles were in the 3–4.5 µm range. Based on the results, it could be concluded that the application of a high-energy ball milling process resulted in the formation of smaller particles, which were better dispersed in the polyol system. Moreover, due to the modification of the filler with functionalized MMT, some functional groups (amine, silane groups) were incorporated into the polyol system. Therefore, the filler particles could react with the polyol system, which prevented their agglomeration. Contrary, unmodified SO filler was not functionalized effectively, and the filler particles tended to agglomerate together instead of interacting with the PUR system.

**Figure 4:**
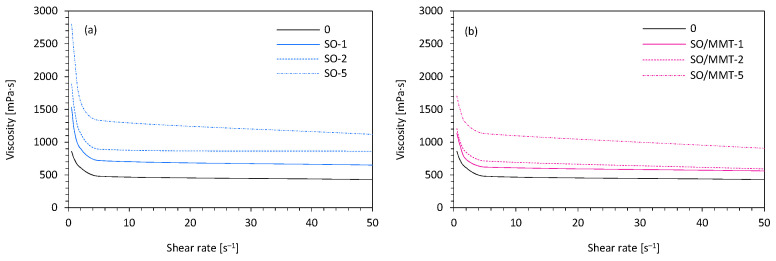
The viscosity of polyol systems containing (**a**) SO filler, and (**b**) MMT-modified SO filler.

The size and distribution of filler particles affected the viscosity of the polyol system (Figure 4). The viscosity of the reference PUR system (without SO filler addition) measured at the share rate of 0.5 s^−1^ was ~900 mPa·s. After adding SO filler into the PUR system, the viscosity increased with increasing filler content. On the addition of 1 and 2 wt.% of SO filler, the viscosity increased relatively slowly; however, after that, the value increased more significantly. Comparing to the neat PUR system, on the addition of 1 and 2 wt.%, the viscosity increased to ~1400 and ~1900 mPa·s, respectively. When the loading of SO filler was up to 5 wt.%, the viscosity increased to 2800 mPa·s. A similar trend was observed for PUR systems with the addition of MMT-modified SO filler; however, compared with PUR systems containing unmodified SO filler, the obtained viscosities were lower. On the addition of 1, 2, and 5 wt.% of MMT-modified SO filler, the viscosity increased to ~1100, ~1250, and ~1790 mPa·s, respectively. This indicated that the filler modification avoided the filler agglomeration and resulted in a better distribution of the filler in the PUR systems. The addition of unmodified SO filler caused the formation of agglomerates, which increased the viscosity of the overall mixture.

### 2.2. PUR Composite Characterization

**Figure 5:**
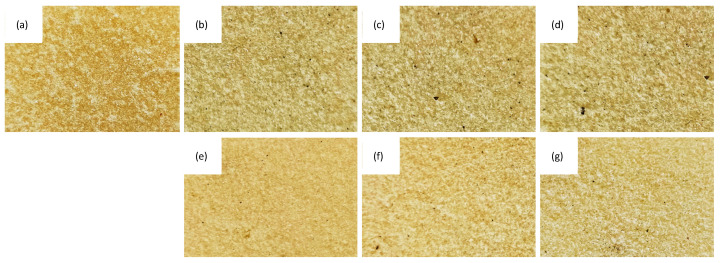
Optical images of the external surface of (**a**) polyurethane (PUR)-0, (**b**) PUR-SO-1, (**c**) PUR-SO-2, (**d**) PUR-SO-5, (**e**) PUR-SO/MTM-1, (**f**) PUR-SO/MMT-2, (**g**) PUR-SO/MMT-5.

**Figure 6:**
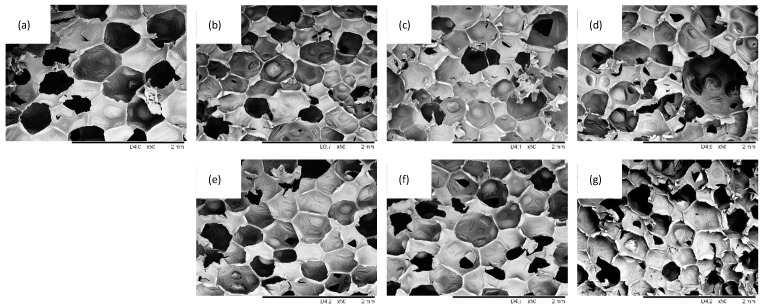
SEM images of (**a**) PUR-0, (**b**) PUR-SO-1, (**c**) PUR-SO-2, (**d**) PUR-SO-5, (**e**) PUR-SO/MTM-1, (**f**) PUR-SO/MMT-2, (**g**) PUR-SO/MMT-5.

**Figure 7:**
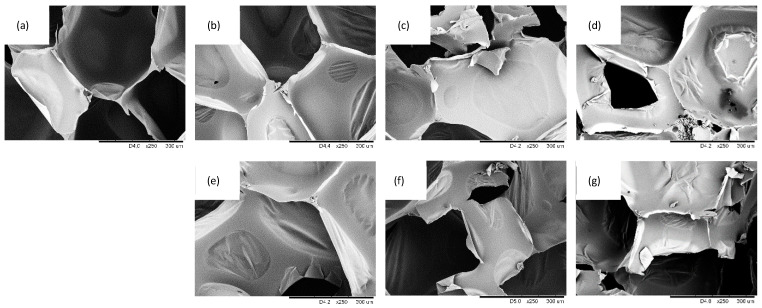
SEM images of (**a**) PUR-0, (**b**) PUR-SO-1, (**c**) PUR-SO-2, (**d**) PUR-SO-5, (**e**) PUR-SO/MTM-1, (**f**) PUR-SO/MMT-2, (**g**) PUR-SO/MMT-5.

Optical images and SEM images of PUR composites filled with different SO filler contents are presented in Figure 5, Figure 6 and Figure 7. Reference PUR foams exhibited well-developed, hexagon shape cell structures. The incorporation of SO filler affected the morphology of PUR composites. In general, the addition of 1 wt.% of SO filler improved the morphology of PUR composites—the overall structure was uniform with a great number of regular closed cells. With the addition of higher loading of SO fillers, the structure of the PUR composites became less uniform, while the uniformity of the cells became poor. Moreover, some particles of the SO filler were visible inside the cells of the PUR composites. This indicated that SO filler tended to agglomerate, and the particles were not well-built into the structure, deteriorating the foaming process and leading to the deterioration of the structure of PUR composites. The closed-cell content decreased from 87.7% (for PUR-0) to 87.0% and 86.1% for PUR composites filled with 1 and 2 wt.% of SO filler, respectively. A more regular structure of PUR composites was observed, with the addition of MMT-modified SO filler. By increasing the filler content, the cellular structure of PUR composites became less uniform; however, when compared with PUR composites filled with unmodified SO filler, the changes were not as significant. With the addition of each amount of MMT-SO filler, the overall morphology of PUR composites was well-preserved, while the content of closed cells was relatively high (>86%). Therefore, it may be concluded that the modification of SO filler with MMT resulted in better compatibility between SO filler and PUR matrix, which resulted in the formation of a more stable structure of PUR composites. This may be connected with higher cross-linking of PUR composites filled with MMT-treated SO filler. Functional groups of hydroxyl-modified MMT may form a chemical bonding with the isocyanate groups during the synthesis of PUR, which resulted in improved cellular structures relative to unmodified PUR foams. The hydroxyl groups of MMT-modified SO filler may participate in the PUR synthesis during the foaming process, enhancing the strength of the cell wall and reducing the occurrence of broken pores. Hence, the cellular structure of reinforced PUR composites may be more stable.

**Figure 8:**
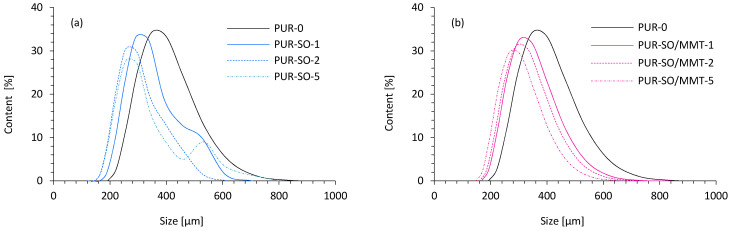
The cell size distribution of PUR composites reinforced with (**a**) unmodified SO filler, and (**b**) MMT-modified SO filler.

According to the results presented in Figure 8, with the increase of SO filler, an average diameter of the cells decreased for PUR composites filled with 1, 2, and 5 wt.% of unmodified SO filler. It could be concluded that when the content is 1 and 2 wt.%, the SO filler could be properly dispersed in PUR systems and could effectively act as nucleating centers of the cells, increasing the number of the cells per unit area. Therefore, the diameter of the cells was decreased. With the addition of a higher content of SO filler, such as 5 wt.%, some agglomeration may occur, which was related to the high surface energy of the SO filler. Agglomerated particles of the SO filler may result in a fraction of the cells, increasing the number of the broken cells. Due to the coalescence of the broken cells, the distribution of their size became less uniform. A similar trend was observed in the case of PUR composites filled with an MMT-modified SO filler; however, such reinforced PUR composites were characterized by smaller cells and more uniform distribution of the cells when compared to PUR filled with an unmodified SO filler. Based on this, it may be concluded that the chemical interaction between MMT-modified SO filler and PUR matrix resulted in more homogenous dispersion of the filler in the PUR system and improved the overall morphology of PUR composites relative to PUR composites filled with unmodified systems.

**Figure 9:**
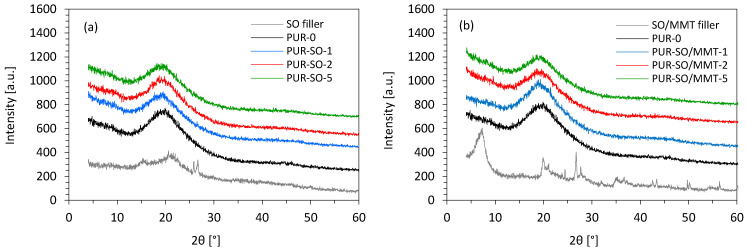
XRD patterns of PUR composites reinforced with (**a**) unmodified SO filler, and (**b**) MMT-modified SO filler.

X-ray diffraction (XRD) analysis was used to determine the effect of SO fillers on the macro- and microstructure changes of PUR composites. Figure 9 shows the XRD patterns of unmodified SO filler, MMT-modified SO filler, and PUR composites. Unmodified SO filler had four peaks at 2θ = 16°, 19–25°, which corresponded to the diffractions of amorphous cellulose II and amorphous cellulose I, respectively [49,50]. Some impurities in the SO filler might have driven two sharp peaks at 2θ = 26° and 27°. After the modification of the SO filler with MMT, some new peaks appeared at the XRD diffractogram and confirmed the characteristics of the montmorillonite type clay reported in previous works [51,52]. More specifically, the MMT-modified SO filler exhibited diffraction peaks at 2θ = 7°, 20°, 25°, 32°, and 60°, which corresponded to montmorillonite type clay [51]. The peak at 2θ = 62° indicated that MMT had a dioctahedral structure [52]. The other peaks were impurities corresponding to quartz. For the PUR-0, wide diffraction from 15–25° with a maximum peak appeared at approximately 21°. For PUR composites, the inclusion of SO fillers did not change the XRD patterns significantly. Introducing unmodified SO filler and MMT-modified SO filler into the PUR matrix decreased the intensity after the incorporation of 1, 2, and 5 wt.% of SO fillers. The intensity of the peak decreased from 551 a.u. (for PUR-0) to 504, 455, and 449 a.u. with the incorporation of 1, 2, and 5 wt.% of unmodified SO filler. A similar trend was observed in the case of PUR composites reinforced with MMT-modified SO filler—on the addition of 1, 2, and 5 wt.% of MMT-modified SO filler, the intensity of the peak decreased to 543, 477, and 470 a.u., respectively. This could be connected with the fact that the introduction of SO fillers disrupted the originally uniform PUR structure and made the PUR composites more amorphous [53]. This effect was more prominent in the case of PUR composites containing unmodified SO filler. When compared with PUR composites containing unmodified SO filler, the introduction of MMT-modified SO filler resulted in the formation of more uniform composites; thus, the intensity was slightly increased.

The apparent density of PUR composites is presented in Table 1. In general, the apparent density increased when the SO fillers were added. This may be connected with the higher viscosity of the initial PUR systems and the molecular weight of SO fillers. Additionally, the changes in apparent density may be considered negligible taking into consideration that the apparent density of PUR composites depended on environmental conditions such as temperature and moisture. The addition of SO fillers affected the thermal conductivity (λ) of PUR composites. In both cases, on the addition of unmodified and MMT-modified SO fillers, the value of thermal conductivity slightly increased. On the addition of unmodified SO filler, the value of λ increased from 0.025 Wm^−1^ K^−1^ (for PUR-0) to 0.026, 0.028, and 0.035 Wm^−1^ K^−1^, respectively, while on the addition of MMT-modified SO filler, the value increased from 0.025 to 0.030 Wm^−1^ K^−1^. In both cases, such an increase in thermal conductivity may be connected with the incorporation of solid particles, which increased the value of λ_solid_. Furthermore, as discussed previously, with increasing the content of SO fillers the number of closed cells was decreased, while the number of broken cells was increased. Taking into consideration that the thermal conductivity of the air (λ = 0.025 Wm^−1^ K^−1^) was higher than that of CO_2_, the thermal conductivity of PUR composites was increased. Most importantly, on the addition of MMT-modified SO filler, the changes in λ were less noticeable than in the case of PUR composites containing unmodified SO filler. This may be connected with a more uniform structure of PUR composites reinforced by MMT-modified SO filler and a higher number of closed cells.

**Figure 10:**
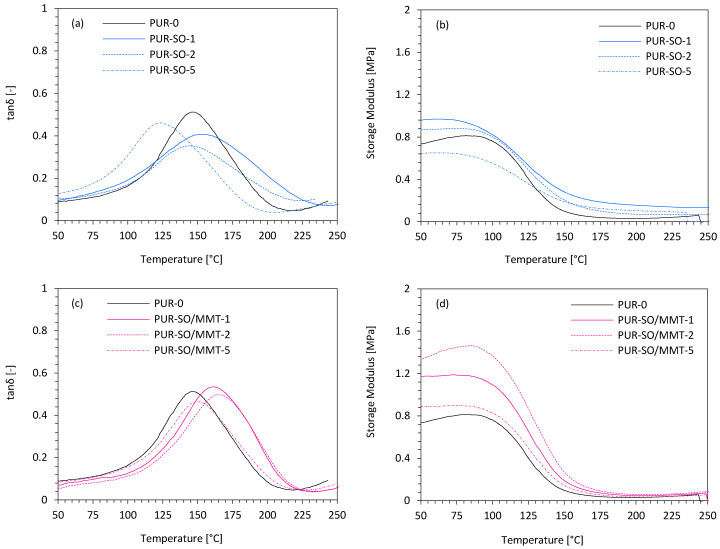
Dynamic mechanical properties of PUR composites—(**a**,**b**) glass transition temperature, and (**c**,**d**) storage modulus.

The results of tanδ and storage modulus (E’) of PUR composites with SO fillers are presented in Figure 10. When compared with reference PUR-0, the value of E’ dramatically increased after the incorporation of MMT-modified SO filler indicating the reinforcing character of the filler. It was concluded that the functional groups of MMT-modified SO filler react with isocyanate groups increasing the cross-linking density of PUR composites. As a result, the PUR chains were connected to each other, and the mobility of the PUR structure was reduced, which, in turn, increased the stiffness of the PUR composites. A slight deterioration in E’ was observed for PUR composites filled with unmodified SO filler. This may be connected with the poor cellular structure of such-filled PUR composites (see Figure 6). Most importantly, the value of E’ was still higher than that of the reference PUR-0, without the filler addition. The glass transition temperature (T_g_) of the PUR soft segments referred to the peak of the tanδ curve. The value of T_g_ of PUR-0 was 147 °C. After the incorporation of 1 and 2 wt.% of unmodified SO filler, the value of T_g_ increased slightly to 156 and 148 °C, respectively. With increasing the content of unmodified SO filler to 5 wt.%, the value of T_g_ decreased to 125 °C. This indicated that the addition of unmodified SO filler in a higher concentration resulted in the formation of a more flexible structure in the PUR composites with a greater number of open cells. Significant improvement in T_g_ was observed after the incorporation of MMT-modified SO filler—depending on the amount of MMT-modified SO filler, the value of T_g_ increased from 153 to 162 °C. Such improvement may be connected with the fact that MMT-modified SO filler could effectively reduce the mobility of PUR segments as a result of chemical bonding of functional groups of MMT with PUR macromolecules. Therefore, the value of T_g_ was increased.

**Figure 11:**
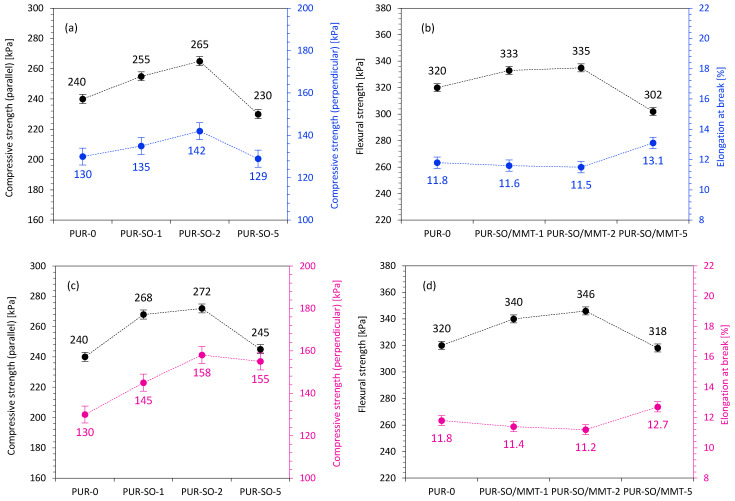
Mechanical performances of PUR composites reinforced with (**a**,**b**) unmodified SO filler and (**c**,**d**) MMT-modified SO filler.

The mechanical performances of the two kinds of PUR composites were examined, and the results are shown in Figure 11. According to the presented results, the compressive strength of the unmodified PUR was 240 kPa. When the PUR composites were reinforced with 1 and 2 wt.% of unmodified SO filler, the compressive strength increased by ~6 and ~10%, respectively. After that, with increasing the SO filler content up to 5 wt.%, the compressive strength was reduced compared with PUR-0 the value decreases by ~4%. This may be connected with the agglomeration of the SO filler in PUR composites. As discussed previously, the addition of SO filler increased the viscosity of the PUR systems, which resulted in non-uniform mixing of SO filler and its entanglement. Therefore, the uniformity of the composites was decreased, which resulted in the deterioration of mechanical performances of PUR composites at higher loading of the filler. For PUR composites reinforced with MMT-modified SO filler, the compressive strength exhibited a similar trend; however, the obtained values were higher compared with PUR composites reinforced with unmodified SO filler. More significant improvement in compressive strength was observed on the addition of 1 and 2 wt.% of MMT-modified SO filler—when compared to PUR-0, the value increased by ~13 and ~15%, respectively. Reinforcement with 5 wt.% of MMT-modified SO filler resulted in a decreased compressive strength compared with 1 and 2 wt.% filler reinforcement. This may be connected with the agglomeration of SO filler particles, which may act as stress concertation points reducing their reinforcing effect and deteriorating the mechanical strength of PUR composites. An analog trend was observed for the flexural strength of the PUR composites. In both cases, the variation trend was similar to the compressive strength dependency. With the addition of 1 and 2 wt.% of unmodified SO filler, the flexural strength increased by ~4 and ~5%; however, after adding 5 wt.% of SO filler, the flexural strength decreased simultaneously. In the case of PUR composites reinforced with 1 and 2 wt.% of MMT-modified SO filler, the flexural strength increased by ~8 and ~10%, respectively, and then decreased slightly for PUR composites reinforced with 5 wt.% of MMT-modified SO filler. When comparing both series of PUR composites, it could be concluded that PUR composites reinforced with MMT-modified SO filler exhibited improvement in mechanical performances due to the reinforcement effect of the rigid MMT, which effectively stiffened the molecular mobility of PUR chains and exhibited a good reinforcing effect.

**Figure 12:**
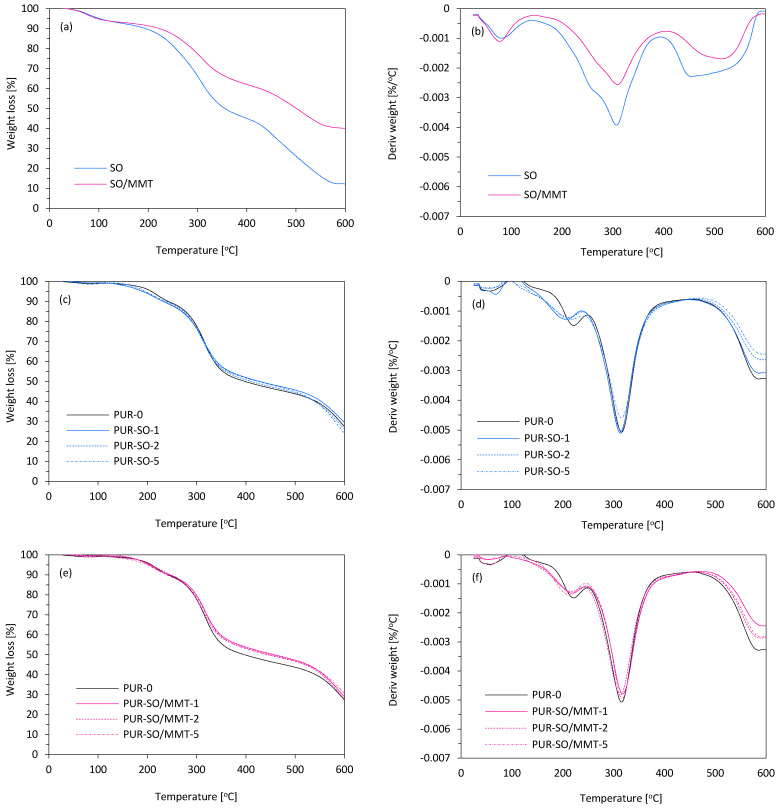
Thermogravimetric analysis of (**a**,**b**) unmodified and MMT-modified SO filler, (**c**,**d**) PUR composites reinforced with unmodified SO filler, and (**e**,**f**) PUR composites reinforced with MMT-modified SO filler.

The thermogravimetric analysis of SO and SO/MMT fillers and PUR composites with their addition was also carried out. The results of the weight loss analysis as a function of temperature (TGA) and mass derivative (DTG) are shown in Table 2 and Figure 12.

Based on the obtained thermograms, the following parameters were determined: the temperature of 5, 10, and 50% weight loss (T_5%_, T_10%_, T_50%_); the temperature of the maximum rate of decomposition of thermal degradation of the material (T_max_); the maximum degradation rate (V_max_); and the char residue after degradation at 600 °C. Table 2 presents the results of the TG and DTG curves analysis obtained during the TGA analysis of fillers and PUR composites with their addition. The T_5%_ temperature was often related to the temperature at which volatile substances evaporated [54,55,56]. This was the case with fillers for which the temperature of the initial decomposition of SO and SO/MMT was 101 and 95 °C, respectively, which was related to the evaporation of bound or absorbed water. Furthermore, along with the fillers, we also introduced the so-called secondary components, which also evaporated at relatively low temperatures [57]. When analyzing the further course of the thermal decomposition of fillers, a significant difference in the thermal stability of sage with layered aluminosilicate compared to pure sage could be noticed. The T_50%_ temperature was much higher for SO/MMT (501 °C) than for SO (355 °C). The higher stability of the SO/MMT system was also confirmed by the remaining content of charred dust obtained at 600 °C. In the case of PUR foams, the temperature T_10%_ was often regarded as the temperature of the onset of thermal degradation. T_10%_ values ranged from 235–241 °C. The addition of fillers did not cause large changes in T_10%_ compared to the reference PUR-0. This proved that all composites with fillers were characterized by similar susceptibility to thermal degradation. It was known that the onset of thermal degradation took place mainly in the segments of rigid PUR macromolecules [58]; therefore, it could be concluded that the addition of neat natural fillers, and in combination with MMT, caused slight changes in the structure of these segments in the obtained polymer composites. Probably, in the modified foams, a slightly larger amount of urea bonds formed during the reaction of water with fillers and -NCO groups of the isocyanate. When analyzing the further thermal decomposition of the foams, it could be noticed that the T_50%_ reacted differently for filled composites compared to PUR-0. While the addition of SO improved the thermal stability compared to the reference PUR-0, there was a downward trend as a function of the filler content; the addition of the SO/MMT mixture significantly improved the resistance to high temperatures. In the case of the composite containing 5 wt.% of SO/MMT, T_50%_ was 457 °C, while for PUR-0, it was 397 °C, which proved a very high stabilizing effect of MTM at high temperatures. The residue of the char after combustion, determined at the temperature of 600 °C, depended on the type and content of the filler. 1 wt.% SO addition slightly increased the content of the char residue in comparison with PUR-0, which could be caused by lower porosity and higher apparent density of the PUR composites. However, with the increase in the content of filler of natural origin with very low thermal stability, this parameter for the PUR composites decreased. The results were slightly different for PUR composites with the addition of SO/MMT, for which there were an upward trend and the highest (31%) content of char residue after the thermal decomposition process, which confirmed the stabilizing effect of MMT. Based on the DTG curves, the characteristic peak for PUR was visible, which showed the maximum rate of decomposition at T_max_ (315–317 °C). A multiplet band was formed as a result of thermal degradation of urethane and urea bonds occurring in rigid segments, and as a result of the decomposition of flexible segments resulting from a mixture of polyols [57,58,59]. Another band above the temperature of 450 °C was created as a result of the thermolysis of organic residues. These compounds remained after the degradation from the earlier stage and were the remains of the rigid and flexible segments [59,60].

The burning behavior and flame-retardant properties of PUR composites were determined using the cone calorimetry method. The results obtained during the study including the ignition time (IT), the peak rate of heat release (pHRR), the total smoke release (TSR), the total heat release (THR), the average yield of CO and CO_2_ (COY and CO_2_Y), and the limiting oxygen index (LOI) are summarized in Table 3.

**Figure 13:**
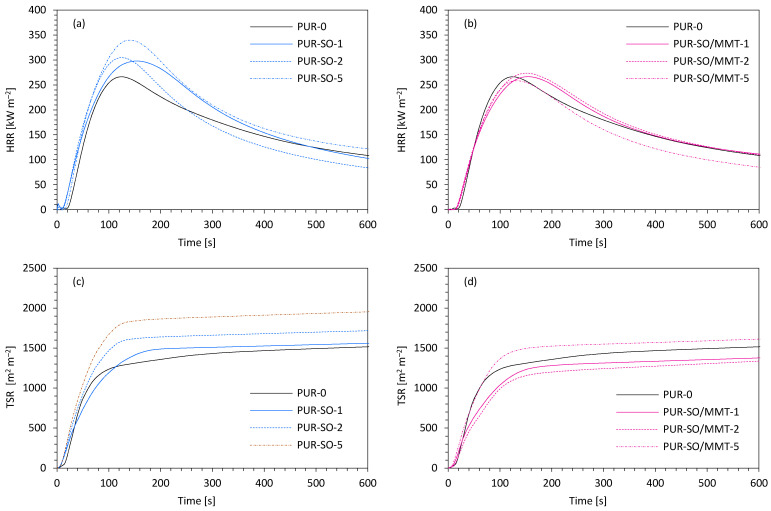

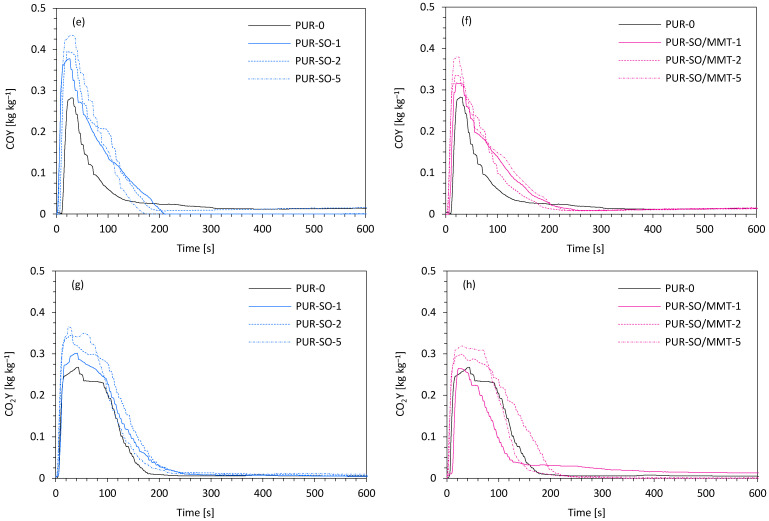
The results of the cone calorimeter test—(**a**,**b**) the peak rate of heat release (pHRR), (**c**,**d**) the total smoke release (TSR), (**e**,**f**) the average yield of CO, and (**g**,**h**) the average yield of CO_2_.

When comparing the modified PUR composites to the reference PUR-0, it could be noticed that the modifications affected the ignition time (IT) slightly. In the case of a higher content of SO filler, a slight reduction of this time could be seen. Whereas analyzing samples additionally modified with MMT, they showed higher values than in the case of the addition of unmodified SO filler. However, in all cases, the value of IT oscillated between 3 and 5 s. The flame intensity was measured by the value of heat peak release (pHRR), which was corresponding to the release of low molecular weight compounds, such as isocyanate, olefins, or amines. As it can be noticed in Figure 13a,b, all analyzed foams exhibited one peak of this parameter. When compared with PUR-0, the value for foams with the sage addition increased from 265 kW m^−2^ to even 340 kW m^−2^ for PUR-SO-5. However, when comparing foams with the addition of MMT modified sage to the reference one, lower values were observed—259 kW m^−2^, 254 kW m^−2^, and 263 kW m^−2^ for PUR-SO/MMT-1, PUR-SO/MMT-2, and PUR-SO/MMT-5, respectively.

When analyzing the effect of modifiers on the total smoke release (TSR) presented in Figure 13c,d, it can be noticed that the addition of SO filler increased this parameter proportionally to the amount of the modifier. While comparing the effect of MMT-modified SO filler, the values of the TSR parameter were lower than for PUR-0 (1490 m^2^ m^−2^) in the case of PUR-SO/MMT-1 (1380 m^2^ m^−2^) and PUR-SO/MMT-2 (1330 m^2^ m^−2^), and a higher value which was 1550 m^2^ m^−2^ in the case of PUR-SO/MMT-5. The incorporation of SO fillers affects the total heat release (THR). By analyzing the impact of fillers, similar conclusions as in the case of the TSR parameter could be drawn. The addition of unmodified SO filler increased the THR value along with the increase in the filler content. When comparing PUR composites with the addition of MMT-modified SO filler to the reference one, it could be observed that in the case of PUR-SO/MMT-1 and PUR-SO/MM-2 this parameter was reduced (respectively 21.1 MJ m^−2^ and 21.0 MJ m^−2^), while, in the case of PUR-SO/MMT-5, this value was higher compared to the reference foam PUR-0 (21.7 MJ m^−2^) and amounts to 21.9 MJ m^−2^. The inclusion of sage fillers also affected the parameter related to the toxicity of foams, which was the ratio of carbon monoxide (CO) to carbon dioxide (CO_2_). Generally, the higher value of this ratio indicated incomplete combustion of PUR composites and a greater amount of toxic smoke. On analyzing the data in Figure 13e–g and h, it could be noticed that for all foams with the addition of fillers, both those with unmodified and MMT-modified SO filler, the COY/CO_2_Y ratio was greater than of the PUR-0. Regarding the limiting oxygen index (LOI), it could be observed that the value of this index decreased with the increase in the filler content in the case of unmodified SO filler. All the LOI values of the foams with unmodified sage were lower than in the case of the reference foam (20.4%), while in the case of MMT-modified sage the LOI reached higher values, which were 21.1%, 21.3%, and 20,8%, respectively, for PUR-SO/MMT-1, PUR-SO/MMT-2, and PUR-SO/MMT-5. Based on the obtained results, it could be concluded that the use of unmodified SO filler deteriorated the flammability properties of the obtained PUR composites. In the case of MMT-modified SO filler, the best results were achieved for PUR-SO/MMT-2 and partially for PUR-SO/MMT-1. In the case of PUR composites containing 2 wt.% and 1 wt.% of this filler, a slight improvement in HRR, TSR, THR, and LOI parameters was observed. Additionally, when it came to PUR-SO/MMT-1, there was also an improvement in the ignition time. A drawback may be an increased COY/CO_2_Y ratio.

## 3. Materials and Methods

### 3.1. Materials

- Isocyanate compound—polymeric diphenylmethane diisocyanate, pMDI (Purocyn B; Purinova Company, Bydgoszcz, Poland);
- Polyol compound—polyether polyol (Stapanpol PS-2352, Stepan Company, Northfield, IL, USA);
- Catalysts—potassium octoate (Kosmos 75) and potassium acetate (Kosmos 33; Evonik Industry, Essen, Germany);
- Surfactant—silicone-based surfactant (Tegostab B8513, Evonik Industry, Essen, Germany);
- Blowing agent—pentane and cyclopentane (Sigma-Aldrich Corporation, Saint Louis, MO, USA);
- Surface modified montmorillonite clay, 15–35 wt.% octadecylamine, 0.5–5 wt.% aminopropyltriethoxysilane (Sigma-Aldrich Corporation, Saint Louis, MO, USA);
- Salvia residue (local company, Lodz, Poland);
- Montmorillonite clay, Nanomer^®^ contains 15–35 wt.% octadecylamine, 0.5–5 wt.% aminopropyltriethoxysilane (Merck, Darmstadt, Germany).

### 3.2. Methods and Instruments

The viscosity of the polyol systems was evaluated using a Viscometer DVII+ (Brookfield, Germany) in the function of a shear rate according to ISO 2555. The measurement was performed at ambient temperature.

The apparent density of the analyzed foams was measured accordingly to the standard ASTM D1622 (equivalent to ISO 845). The density was tested on five samples of each foam and expressed as an average.

Cell size distribution and foam morphology were examined on the basis of the cellular structure pictures of foams taken using JEOL JSM-5500 LV scanning electron microscopy (JEOL LTD, Akishima, Japan). The microscopic research was carried out in a high-vacuum mode and at the accelerating voltage of 10 kV. Foam samples were scanned parallel to the foam growth direction. 

XRD analysis was conducted with a diffractometer DRON-7 (Research and Production Enterprise Bourevestnik, Russia) using Cu Kα radiation, for the anode voltage of 30 kV, anode current of 12 mA, detector movement step of 0.02 mm, and diffraction angle up to 60° for SO filler and PUR composites reinforced with unmodified SO filler, and up to 80° for MMT-modified SO filler and PUR composites reinforced with MMT-modified SO filler. The ICDD database was used for the XRD patterns analysis.

A three-point bending test was carried out accordingly to the standard ASTM D7264 (equivalent to ISO 178) using a Zwick Z100 Testing Machine (Zwick/Roell Group, Germany). The analyzed samples were bent with a speed of 2 mm min^−1^. For each series of foams, at least five measurements were made. Obtained flexural stress at the break results for each sample was expressed as a mean value and averaged.

The compressive strength (σ_10%_) of the foams was determined accordingly to the standard ASTM D1621 (equivalent to ISO 844). The measurement was conducted using a Zwick Z100 Testing Machine (Zwick/Roell Group, Germany) with a load cell of 2 kN and a speed of 2 mm min^−1^. The compression strength was examined as a ratio of the load causing 10% deformation of samples cross-section in both parallel and perpendicular direction to the square surface. The compressive strength was measured in five samples of foam (8 cm × 8 cm × 5 cm) and expressed as an average.

Dynamic mechanical analysis (DMA) was determined using ARES Rheometer (TA Instruments, New Castle, DE, USA). Torsion geometry was used for samples with a thickness of 2 mm. Measurements were examined in a temperature range of 20–250 °C at a heating rate of 10 °C min^−1^, using a frequency of 1 Hz and applied deformation at 0.1%.

The thermal stability of the foams was analyzed using a Mettler Toledo thermogravimetric analyzer TGA/DSC1. A thermal decomposition examination was conducted in an inert gas atmosphere (flow 50 mL min^−1^) and in the temperature range between 25 and 600 °C (heating rate 10 °C min^−1^). The measurement included an analysis of the mass change as a function of temperature during thermal decomposition of the polyurethane foams. The initial temperatures of the following decomposition stages were noticed and designated as T_5%_, T_10%_, T_50%_. These temperature values corresponded to the percentage of weight loss.

The burning behavior and flame-retardant properties of the foams were analyzed using a cone calorimeter, according to the standard ISO 5660 in S.Z.T.K. TAPS (Maciej Kowalski Company, Saugus, Poland). The measurement for each foam was repeated on three samples and averaged. Each specimen with dimensions of 10 cm × 10 cm × 5 cm was wrapped with aluminum foil and burned at an external heat flux of 35 kW m^−2^. The parameters were recorded during the time.

### 3.3. Polyurethane Composite Synthesis

**Figure 14:**
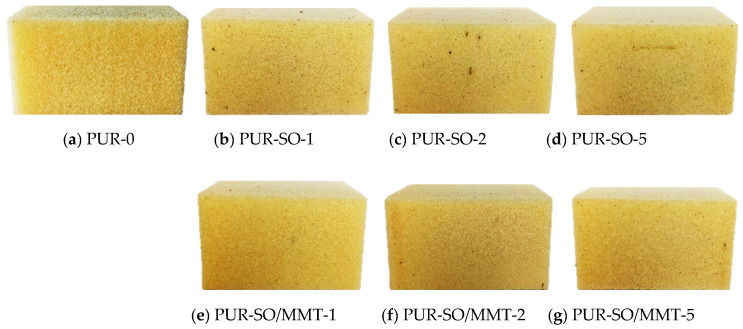
Optical images of PUR composites prepared in this study—(**a**) PUR-0, (**b**) PUR-SO-1, (**c**) PUR-SO-2, (**d**) PUR-SO-5, (**e**) PUR-SO/MMT-1, (**f**) PUR-SO/MMT-2, (**g**) PUR-SO/MMT-5.

Before being added to the polyol system, the SO filler was mechanically ground and sieved using a 100 µm sieve. In the next step, the SO filler was mixed with MMT powder (1:2% *w/w*) and milled using a high-energy ball milling process (30 min, 2000 rpm, ball to powder ratio = 12:1). SO fillers were used as reinforcing fillers of PUR composites. The effect of filler content on the properties of PUR composites was evaluated. Therefore, the SO fillers were prepared in weight compositions of 1, 2, and 5 wt.%. PUR composites were produced through a process of component addition. Firstly, a polyol system consisting of polyol, water, catalysts, and the blowing agent was placed in the beaker. A calculated amount of SO filler (1, 2, or 5 wt.%) was added to the polyol system. Such prepared composition was mixed vigorously using a mechanical stirrer (2000 rpm, 60 s). In the next step, an isocyanate component was added to the polyol system, and the mixture was mixed for another 60 s (2000 rpm). PUR composites were left to expanse freely and cure at room temperature for 24 h. The formulas of PUR composites prepared in this study are presented in Table 4. Optical images of PUR foams prepared in this study are presented in Figure 14.

## 4. Conclusions

Rigid polyurethane (PUR) foams reinforced with 1, 2, and 5 wt.% of salvia filler (SO filler) and MMT-modified salvia filler (MMT-modified SO filler) were produced in this study. The impact of 1, 2, and 5 wt.% of SO filler and MMT-modified SO filler on the morphological, chemical, and mechanical properties of PUR composites were examined. In both cases, the addition of 1 and 2 wt.% of SO fillers resulted in the synthesis of PUR composites with improved physicomechanical properties, while the addition of 5 wt.% of SO fillers resulted in the formation of PUR composites with a less uniform structure and, therefore, some deterioration in their physicomechanical performances. Moreover, the results showed that the modification of SO filler with MMT improved the interphase compatibility between filler surface and PUR matrix. Therefore, such reinforced PUR composites were characterized by a well-developed closed-cell structure and improved mechanical, thermal, and flame-retardant performances. For example, when compared with reference foam, the addition of 2 wt.% of MMT-modified SO filler resulted in the formation of PUR composites with greater compressive strength (~15% increase) and improved flexural strength (~10% increase). The glass transition temperature increased from 147 to 157 °C, while the storage modulus increased by ~43%. The PUR composites were characterized by better thermal stability as well as improved flame retardancy—e.g., the peak rate of heat release (pHRR) decreased from 265 to 250 kW m^−2^, the total smoke release (TSR) decreased from 1490 to 1330 m^2^ m^−2^, while the limiting oxygen index (LOI) increased from 20.4 to 21.3%. The results presented in the current study confirmed that the addition of a proper amount of MMT-modified SO filler can be an effective, inexpensive, and environmentally-friendly approach to the synthesis of PUR composites with improved physicomechanical, thermal, and fire-retardant performances.

## Figures and Tables

**Table 1:** Selected properties of PUR composites—cell size, closed cell content, apparent density, and thermal conductivity.

	Cell Size (µm)	Closed Cell Content (%)	Apparent Density (kg m^∓3^)	Thermal Conductivity (W m^−1^K^−1^)
PUR-0	347 ± 6	87.4 ± 0.6	35.4 ± 0.8	0.025
PUR-SO-1	338 ± 6	87.0 ± 0.5	36.2 ± 0.9	0.026
PUR-SO-2	296 ± 5	86.1 ± 0.7	36.9 ± 0.6	0.028
PUR-SO-5	296 ± 4	83.0 ± 0.9	37.6 ± 0.6	0.035
PUR-SO/MMT-1	310 ± 5	88.2 ± 0.6	35.7 ± 0.8	0.025
PUR-SO/MMT-2	289 ± 6	88.0 ± 0.7	36.1 ± 0.7	0.027
PUR-SO/MMT-5	285 ± 7	86.5 ± 0.7	37.0 ± 0.8	0.030

**Table 2:** The results of the thermogravimetric analysis (TGA) analysis.

Sample	T_5%_ (°C)	T_10%_ (°C)	T_50%_ (°C)	Char Residue (at 600 °C)	T_max_ (°C)	V_max_ (%/min)
SO	101	195	355	12.2	309	0.0025
SO/MMT	95	223	501	40.1	307	0.0039
PUR-O	207	241	397	27.6	315	0.0051
PUR-SO-1	191	231	425	29.6	313	0.0051
PUR-SO-2	189	233	423	26.4	313	0.0050
PUR-SO-5	195	239	419	24.2	315	0.0045
PUR-SO/MMT-1	197	239	443	27.9	317	0.0048
PUR-SO/MMT-2	205	241	449	29.1	315	0.0048
PUR-SO/MMT-5	203	235	457	31.0	317	0.0049

**Table 3:** The results of the cone calorimeter test.

Sample	IT (s)	pHRR (kW m^−2^)	TSR (m^2^ m^−2^)	THR (MJ m^−2^)	COY (kg kg^−1^)	CO_2_Y (kg kg^−1^)	COY/CO_2_Y (-)	LOI (%)
PUR-0	4	265	1490	21.7	0.272	0.266	1.02	20.4
PUR-SO-1	4	280	1550	21.9	0.370	0.311	1.19	20.2
PUR-SO-2	3	292	1650	22.4	0.376	0.342	1.10	20.1
PUR-SO-5	3	340	1890	23.6	0.434	0.363	1.20	19.5
PUR-SO/MMT-1	5	254	1380	21.1	0.312	0.260	1.20	21.1
PUR-SO/MMT-2	4	250	1330	21.0	0.334	0.299	1.12	21.3
PUR-SO/MMT-5	4	272	1550	21.9	0.356	0.313	1.14	20.8

**Table 4:** Chemical formulas of PUR composites prepared in the following study.

System	Compound	Content (wt.% to polyol)			
Polyol system	Polyether polyol (Stepanpol PS2352)	100			
	Blowing agent (pentane/cyclopentane, 1:1, *v*/*v*)	11			
	Surfactant (Tegostab B8513)	2.5			
	Catalyst (Kosmos 33)	6			
	Catalyst (Kosmos 75)	0.8			
	Water	0.5			
	unmodified SO filler	0 (PUR-0)	1 (PUR-SO-1)	2 (PUR-SO-2)	5 (PUR-SO-5)
	MMT-modified SO filler	0 (PUR-0)	1 (PUR-SO/MMT-1)	2 (PUR-SO/MMT-2)	5 (PUR-SO/MMT-5)
Isocyanate system	polymeric diphenyl methane diisocyanate, pMDI (Purocyn B)	160			

## Data Availability

Data sharing not applicable.
